# An update on redox signals in plant responses to biotic and abiotic stress crosstalk: insights from cadmium and fungal pathogen interactions

**DOI:** 10.1093/jxb/erab271

**Published:** 2021-06-10

**Authors:** María C Romero-Puertas, Laura C Terrón-Camero, M Ángeles Peláez-Vico, Eliana Molina-Moya, Luisa M Sandalio

**Affiliations:** 1 Department of Biochemistry and Molecular and Cellular Biology of Plants, Estacion Experimental del Zaidin (EEZ), Consejo Superior de Investigaciones Cientificas (CSIC), Apartado 419, 18080 Granada, Spain; 2 Bioinformatics Unit, Institute of Parasitology and Biomedicine “López-Neyra” (IPBLN-CSIC), Granada, Spain; 3 Universidad de Sevilla and CSIC, Spain

**Keywords:** Abiotic stress, biotic stress, cadmium, fungal pathogens, nitric oxide, reactive nitrogen species, reactive oxygen species, redox signalling

## Abstract

Complex signalling pathways are involved in plant protection against single and combined stresses. Plants are able to coordinate genome-wide transcriptional reprogramming and display a unique programme of transcriptional responses to a combination of stresses that differs from the response to single stresses. However, a significant overlap between pathways and some defence genes in the form of shared and general stress-responsive genes appears to be commonly involved in responses to multiple biotic and abiotic stresses. Reactive oxygen and nitrogen species, as well as redox signals, are key molecules involved at the crossroads of the perception of different stress factors and the regulation of both specific and general plant responses to biotic and abiotic stresses. In this review, we focus on crosstalk between plant responses to biotic and abiotic stresses, in addition to possible plant protection against pathogens caused by previous abiotic stress. Bioinformatic analyses of transcriptome data from cadmium- and fungal pathogen-treated plants focusing on redox gene ontology categories were carried out to gain a better understanding of common plant responses to abiotic and biotic stresses. The role of reactive oxygen and nitrogen species in the complex network involved in plant responses to changes in their environment is also discussed.

## Teaser

Reactive oxygen and nitrogen species and redox signals are at the crossroads of plant responses to abiotic and biotic stress and mediate plant-induced resistance/sensitivity, which mainly depends on the specific interaction.

## Introduction

Plants are routinely confronted with more than one stress either simultaneously or sequentially in the field, where a changeable environment exists, especially in the context of global warming, and where pathogens and herbivores are present (Suzuki *et al.*, 2014). In fact, a study of transcriptome responses to different combinations of stresses in Arabidopsis has shown that plants have evolved to cope with combinations of stresses (Rasmussen *et al.*, 2013). An understanding of specific and common biological and molecular responses of plants to different stresses is crucial for crop resistance in the current environmental context. For this reason, in recent years, large-scale transcriptomic analysis involving microarray, RNA-seq, and metabolomic techniques has been used to study crosstalk between different signalling networks (Cheong *et al.*, 2002; Mhamdi and Noctor, 2016; Cohen and Leach, 2019; Zandalinas *et al.*, 2021). Furthermore, large-scale analysis involving 350 Arabidopsis accessions and various combinations of stresses has highlighted genome-wide associations with plant resistance and has identified target genes related to plant responses to multiple stresses (Thoen *et al.*, 2017). Plant responses to more than one simultaneous stress are complex, with a balance between different pathways being required to enable plant survival (Makumburage *et al.*, 2013; Suzuki *et al.*, 2014; Thoen *et al.*, 2017; Zandalinas *et al.*, 2021). The many recent studies, comprehensive reviews, and special issues of scientific journals on different combinations of abiotic stresses highlight the importance of this topic (Loudet and Hasegawa, 2017; Lawas *et al.*, 2018; Sehgal *et al.*, 2018; Balfagón *et al.*, 2019; Zhou *et al.*, 2019; Peck and Mittler, 2020; Zandalinas *et al.*, 2020, 2021). Interestingly, unique plant responses to combinations of abiotic stresses including heat stress induce specific transcription factor (TF) group patterns, which are not shared with other stress combinations (Zandalinas *et al.*, 2020). A recent exhaustive analysis of up to six combined stresses showed that an increase in the number of stresses negatively correlates with plant growth and survival (Zandalinas *et al.*, 2021).

Combinations of abiotic and biotic stresses, and the ways in which adverse growth conditions affect plant responses to pathogens, have attracted less interest from researchers than combinations of different abiotic stresses. In fact, the variable behaviour and the diverse nature of plant infection mechanisms make it difficult to reach general conclusions. In this review, we evaluate the latest data on crosstalk between plant responses to biotic and abiotic stresses, with particular attention paid to the key regulatory role of reactive oxygen species (ROS), reactive nitrogen species (RNS), and redox signals. Analyses of transcriptomes related to plant responses to single and combined stresses will help to decipher plant responses to biotic and abiotic stresses commonly encountered in the field. The results obtained could be used to improve crop stress tolerance in the future. The relationship between plant hyperaccumulation of metals and pathogen defences, the availability of transcriptomes involving the heavy metal cadmium (Cd), and the presence in these transcriptomes of plant responses to biotic stresses, particularly fungal pathogens, enabled us to gain insights into the possible role of ROS/RNS and redox signals at the crossroads of plant responses to Cd and fungi.

## Crosstalk between plant responses to abiotic and biotic stress

**Fig. 1.**
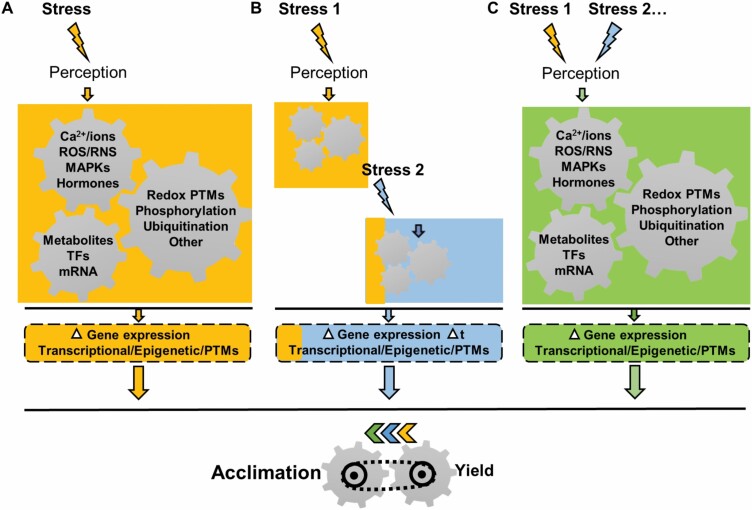
Signal transduction pathways in plant responses to stress. (A) After stress perception, a complex and specific signalling pathway (indicated by the yellow colour) is activated to produce a response leading to plant survival, aimed at achieving a trade-off between acclimation and yield. Signalling pathways involve different factors such as ions/Ca^2+^, reactive oxygen and nitrogen species (ROS/RNS), mitogen-activated protein kinases (MAPKs), hormones, changes in proteins by post-translational modifications (PTMs), and transcription factors (TFs). All these factors need to be integrated to ensure a proper response. (B) Sequential double stress-induced changes are very similar to those observed after the application of the second stress (indicated by the blue colour), although significant signatures from the first stress (indicated by the yellow colour) are also identified. The application of the first stress may also affect the timing of the regulation of specific biological processes related to the second stress. (C) Simultaneous stresses induce unique plant responses to each combination of stresses (indicated by the green colour), which differ from the responses to stresses applied individually.

Protection of plants against disease using abiotic stress treatments previously appeared to be specific to the type of stress encountered and to the behaviour of the pathogen (Rasmussen *et al.*, 2013; Bostock *et al.*, 2014; Zhang and Sonnewald, 2017). Co-expression analysis has revealed a set of gene transcripts with similar profiles of responses to biotic and temperature stresses, mainly associated with the hormones ethylene (ET), jasmonic acid (JA), and/or salicylic acid (SA) (Rasmussen *et al.*, 2013). In a recent genome-wide association mapping study of plant resistance to different biotic and abiotic stresses, genetic correlation analysis showed a strong relationship between plant responses to osmotic stress and root-feeding nematodes (Thoen *et al.*, 2017). Nematodes alter cellular osmotic pressure and plant water potential (Baldacci-Cresp *et al.*, 2015), which link the specific abiotic stress to the plant response to the infection mechanism of these parasites (Atkinson and Urwin, 2012). Heat stress undermines the resistance of tomato to nematodes, although little is known about the underlying mechanism involved (Marques de Carvalho *et al.*, 2015). Insect damage is frequently associated with osmotic stress and drought stress, which appear to strongly overlap in phytohormone-dependent signalling (Ma *et al.*, 2006; Pieterse *et al.*, 2012; Thoen *et al.*, 2017). Following sequential double-stress treatment in Arabidopsis involving a combination of *Botrytis cinerea* infection, *Pieris rapae* herbivory, and drought, changes in the transcriptome profile were very similar to those observed after the application of the second stress, although significant signatures, mainly related to hormones, from the first stress were also identified (Coolen *et al.*, 2016; Fig. 1). The first stress also affected the timing of the regulation of specific biological processes (Coolen *et al.*, 2016). In this case, prior treatment of Arabidopsis with herbivory, but not with drought stress, protected against *B. cinerea* lesion spread, again suggesting that protection is probably treatment-specific (Coolen *et al.*, 2016). Some studies of simultaneous drought/heat and biotic stresses suggest that abiotic stress plays a predominant role, leading to increased plant susceptibility, although the precise mechanisms involved are not fully understood (Luo *et al.*, 2005; Prasch and Sonnewald, 2013; Pandey *et al.*, 2015; Gupta *et al.*, 2020). Other studies suggest that abscisic acid (ABA) reduces plant tolerance to hemibiotrophic and biotrophic pathogens across species (reviewed in Zhang and Sonnewald, 2017). Plant protection against biotic stresses under salt-stress conditions depends on the specific pathogen, with salt-stressed tomato plants being more susceptible to *Oidium neolycopersici* (Kissoudis *et al.*, 2014) and more resistant to *B. cinerea* (Achuo *et al.*, 2006), while salt-stressed barley plants are more resistant to powdery mildew (Wiese *et al.*, 2004). Salt stress has been shown to decrease SA-dependent responses to *Pseudomonas syringae* in tomato plants and to alter negative JA–SA interactions in response to the herbivore *Trichoplusia ni* without affecting resistance to either of these pathogens (Thaler and Bostock, 2004). Temperature changes also affect plant resistance, with low temperatures appearing to prevent gene silencing against viruses (Szittya *et al.*, 2003) and high temperatures contributing to the spread of pathogens such as *Fusarium* (Madgwick *et al.*, 2011). Furthermore, high temperatures induce conformational changes in tobacco mosaic virus R genes, leading to increased susceptibility of tobacco plants (Zhu *et al.*, 2010). On the other hand, high temperatures have been found to contribute to increased resistance of wheat to *Puccinia striiformis* (Carter *et al.*, 2009). This variability in reported results highlights the complexity of biotic and abiotic stress responses, as well as the specific nature of each interaction and situation (Zhu *et al.*, 2010; Prasch and Sonnewald, 2013; Huot *et al.*, 2017). Apart from temperature, other climate-change-related factors, such as increasing CO_2_ emissions, may affect the resistance of crop species (Luck *et al.*, 2011).

## ROS, nitric oxide, and redox signals in plant responses to stress

Data collected over time strongly demonstrate that stress signalling in plants is organized in a complex network mediated by signals, some of which are commonly found in plant responses to abiotic and biotic stresses. Recent research on signalling components, which include calcium (Ca^2+^) and other ions, mitogen-activated protein kinase (MAPK) cascades, hormones, and TFs, and function in biotic/abiotic crosstalk, have been widely reviewed (Fig. 1; Gilroy *et al.*, 2014; Choudhury *et al.*, 2017; Zhang and Sonnewald, 2017; Bai *et al.*, 2018; Zandalinas *et al.*, 2020, 2021). Some of these signalling molecules are ROS/RNS, key molecules that orchestrate crosstalk between plant responses to abiotic and biotic stress. In addition, the two key thiol/disulfide couples, reduced/oxidized glutathione (GSH/GSSG) and cysteine (Cys/CySS), and the ascorbic/dehydroascorbic acid couple (ASC/DHA), as well as a broad range of redox-dependent proteins, lie at the core of the cellular redox state (Bowler and Fluhr, 2000; Baxter *et al.*, 2014; Sandalio *et al.*, 2019; Fichman and Mittler, 2020).

ROS, which are by-products of the plant aerobic metabolism (Inupakutika *et al.*, 2016), have different properties and reactive capacities. They include superoxide (O_2_^.−^) and hydroxyl (·OH) radicals, hydrogen peroxide (H_2_O_2_), and excited singlet oxygen (^1^O_2_). ·OH, which is capable of reacting with virtually all molecules, has a shorter lifetime, while H_2_O_2_ is the most stable and least reactive ROS. The lifetime of O_2_^.−^, which rapidly dismutates to H_2_O_2,_ is shorter than that of H_2_O_2_ and ^1^O_2_, but longer than that of ·OH (Halliwell and Gutteridge, 2007). Plants contain numerous ROS-generating pathways associated with different organelles, which are intimately linked to metabolic pathways and to plant function and development. ROS production in chloroplasts and mitochondria is mainly dependent on photosynthetic electron transport and the mitochondrial electron transport chain (Smirnoff and Arnaud, 2019); ROS production in peroxisomes has been recently reviewed by Sandalio *et al.* (2021).

NADPH oxidase is the principal source of O_2_^.−^ and derived H_2_O_2_ in the apoplast (Suzuki *et al.*, 2011), while peroxidases also contribute to ROS production (Daudi *et al.*, 2012). Although high and uncontrolled levels of ROS can be dangerous, controlled concentrations of ROS play an important role as signals in the regulation of different developmental processes and responses to biotic and abiotic stresses. Antioxidant defences regulate the balance between ROS production and removal, which enables the signalling of these molecules to function. Superoxide dismutase (SOD) disproportionates O_2_^.−^ to H_2_O_2_, and several isoforms of SOD, with different prosthetic metals, are present in all cellular compartments (Gill *et al.*, 2015). H_2_O_2_ is then removed by catalase, the ASC–GSH cycle and peroxiredoxins (Smirnoff and Arnaud, 2019). However, antioxidants do not merely defend against oxidants, but also regulate cellular redox biology. Using the term “ROS-processing systems” rather than “antioxidative systems”, (Noctor *et al.* 2018) suggested that these molecules play a broad role in regulating and transmitting redox-derived signals.

The stability, diffusibility, and selective reactivity of H_2_O_2_ make it an ideal signalling molecule. It can react with sulfur-containing amino acids such as cysteine, leading to its reversible oxidation to sulfenic acid (-SOH; sulfenylation) and sulfinic acid (-SO_2_H; sulfinylation), while excessive ROS accumulation gives rise to an irreversible sulfonic acid (-SO_3_H) derivative (sulfonylation; Young *et al.*, 2019). Sulfenylation and sulfinylation, as well as intra- and inter-molecular disulfide bond formation, are rapid and reversible mechanisms, which regulate protein function, stability, and location (Sandalio *et al.*, 2019; Young *et al.*, 2019). Given their transient nature, these sulfur modifications, which can be reversibly reduced by thioredoxin and glutaredoxin pathways, are regarded as redox switches. The flexibility of these redox circuits favours rapid responses to changes in intracellular redox homeostasis caused by environmental changes, thus regulating metabolic pathways and facilitating signalling networks (Noctor *et al.*, 2018; Sandalio *et al.*, 2019; Young *et al.*, 2019). There is some evidence that ROS production in different organelles, as well as temporary spikes in ROS, leave a specific imprint on the transcriptome response, which can be translated by the cell into specific cellular responses (Rosenwasser *et al.*, 2011; Sewelam *et al.*, 2014).

Nitric oxide (NO) is well known to be a global intra- and intercellular signalling molecule involved in the regulation of an enormous range of plant processes, from development to defence responses to biotic and abiotic stresses (Umbreen *et al.*, 2018; Sánchez-Vicente *et al.*, 2019). Reductive and oxidative mechanisms have been reported to be involved in NO biosynthesis in plants, although this process remains unclear (reviewed in Chamizo-Ampudia *et al.*, 2016; Astier *et al.*, 2018; León and Costa-Broseta, 2020). NO production has been reported in peroxisomes (reviewed in Sandalio *et al.*, 2021), cytosol, mitochondria, and chloroplasts, although the mechanisms involved are not fully understood (León and Costa-Broseta, 2020). NO is also produced in the plasma membrane and apoplast (Stöhr *et al.*, 2001; reviewed in León and Costa-Broseta, 2020). Intracellular levels of NO are regulated by balancing its production, scavenging, and metabolism. NO can react with reduced glutathione (GSH), giving rise to *S*-nitrosoglutathione (GSNO), which in turn is regulated by GSNO reductase (GSNOR) or reacts with O_2_^.−^-producing peroxynitrite (ONOO^−^) (reviewed in Arnaiz *et al.*, 2021). NO levels can be regulated by globins, which are capable of metabolizing NO-producing nitrate (Perazzolli *et al.*, 2006; Becana *et al.*, 2020). The mode of action of NO in plants depends on covalent protein post-translational modifications (PTMs), the best known of which is *S*-nitrosylation (*S*-nitrosation); this PTM involves the formation of a nitrosothiol in a cysteine residue, which can modify the function, location, and stability of a large number of proteins (Romero-Puertas *et al.*, 2013; Feng *et al.*, 2019). Different TFs are targeted by *S*-nitrosylation, which affects their DNA-binding and gene-regulation capacities (Cui *et al.*, 2018, 2020; Imran *et al.*, 2018). NO interacts with most phytohormone metabolisms and/or signalling pathways through the *S*-nitrosylation of key enzymes, and also regulates ROS levels through the *S*-nitrosylation of ROS-producing and ROS-removing enzymes (reviewed in Sandalio *et al.*, 2019). *S*-nitrosylation is a reversible process, which is partly regulated by thioredoxins (Mata-Pérez and Spoel, 2019). Another NO-dependent PTM, whose reversibility remains elusive, is nitration; nitration of proteins and fatty acids affects the functionality of a number of plant proteins and signalling pathways (Mata-Pérez *et al.*, 2017; Arasimowicz-Jelonek and Floryszak-Wieczorek, 2019).

### ROS/RNS and redox signals at the crossroads of plant responses to abiotic and biotic stresses

Virtually all abiotic and biotic stresses induce ROS/RNS production and redox changes, which in turn are connected with MAPK signalling, as well as hormone metabolism and signalling. Signalling mechanisms such as phosphorylation and ubiquitination are regulated by ROS/RNS, as are various TFs, leading to changes in gene expression (Vaahtera *et al.*, 2014; Imran *et al.*, 2018; Sandalio *et al.*, 2019; Siauciunaite *et al.*, 2019). A crucial challenge in redox biology is the identification of sensors that trigger different signalling mechanisms. Interestingly, stomatal movements, which are regulated under various abiotic stresses such as drought, light, ozone, and CO_2_ (Devireddy *et al.*, 2018, 2020; Zhang *et al.*, 2018; Gupta *et al.*, 2020), and are also the entrance point for numerous pathogens (Melotto *et al.*, 2006; Qi *et al.*, 2018), may be involved in crosstalk between abiotic and biotic stresses. Stomatal movements are regulated by a complex signalling network involving ROS/RNS, Ca^2+^ and other ions, channels, and transporters, as well as ABA. One of the first signs of stomatal closure is an increase in ROS in the apoplast and chloroplast (reviewed by Song *et al.*, 2014; Sierla *et al.*, 2016), and NO is also involved in stomatal movements (Van Meeteren *et al.*, 2020). Systemic signalling in plant responses to abiotic stress, which is mediated by ROS mainly derived from NADPH oxidase D [respiratory burst oxidase protein D (RBOHD); Fichman *et al.*, 2019; Fichman and Mittler, 2020; Zandalinas *et al.*, 2020], constitutes another point of crosstalk between abiotic and biotic stresses. MYB30, one of the RBOHD-dependent transcripts regulated during systemic signalling, is involved in plant responses to abiotic and biotic stresses (Mabuchi *et al.*, 2018; Fichman *et al.*, 2020). Cell wall lignification, which is also ROS dependent (Barceló *et al.*, 2004; Pan *et al.*, 2021), may be another point of crosstalk between abiotic and biotic stresses, as various abiotic stresses induce lignin accumulation (Díaz *et al.*, 2001), which is a physical barrier against specific pathogens such as *Verticillium* (Pomar *et al.*, 2004).

Furthermore, a number of studies have analysed ROS/RNS and redox signals at the crossroads of combined abiotic and biotic stresses. Narusaka *et al.* (2004) have reported that treatment of *Arabidopsis thaliana* with copper (Cu) and infection with the necrotrophic pathogens *Alternaria alternata* and *Alternaria brassicicola* cause a significant overlapping of regulation of cytochrome P450 genes, suggesting that common ROS signals trigger similar responses. Down-regulation of O_2_^.−^ and induction of antioxidants are associated with an increase in the sensitivity of tobacco plants to the tobacco mosaic virus at high temperatures, although the mechanisms involved are not well understood (Király *et al.*, 2008). While redox signals are key elements in networks of cross-tolerance to stresses, the role of NO in these networks remains unclear, although its role in plant responses to a single stress has been well documented (Umbreen *et al.*, 2018; Martínez-Medina *et al.*, 2019; León and Costa-Broseta, 2020).

## Crosstalk in plant responses to heavy metals and biotic stress

While some heavy metals (those with density ≥5.0 g cm^−3^), such as iron (Fe), manganese (Mn), and Cu, are essential elements needed for plants to achieve normal metabolism and to carry out physiological processes, other heavy metals, such as Cd, mercury (Hg), chromium (Cr), and the metalloid arsenic (As), are toxic even at low doses (Clemens and Ma, 2016; Terrón-Camero *et al.*, 2019). Nevertheless, essential heavy metals may be toxic to plants at high concentrations, and excessive availability may result from global warming effects such as drought, high temperatures, and flooding. Currently, soil contamination with heavy metals poses a potential threat to the environment and to agriculture, and therefore to human health. The main sources of heavy metals in agricultural soils are anthropogenic activities such as wastewater irrigation from sewage sludge, limestone amendments, and application of inorganic fertilizers (Cao *et al.*, 2016; Clemens and Ma, 2016). Heavy metals/metalloids also occur naturally in sediment deposits in, for example, soil and water (Peralta *et al.*, 2020).

Apart from the risk of sudden pollution spills, plants growing in contaminated soils are already under threat and are likely to face other types of stress, particularly biotic stresses. Heavy metals therefore make for an interesting in-depth case study of crosstalk between abiotic and biotic stresses. It has been suggested that several plant species even capture high concentrations of metals from the soil as a defence mechanism against herbivores and pathogens (Poschenrieder *et al.*, 2006; Llugany *et al.*, 2019). These authors have identified at least five different modes of action induced by metals to counter biotic stress: (i) phytosanitary actions, as various metals are widely used as fungicides, which are detrimental to pathogen and herbivore growth (reviewed in Morkunas *et al.*, 2018); (ii) metal therapy, as metals can activate defence signals to protect the plant against pathogens; (iii) possible trade-offs, whereby a metal defence strategy could save energy for organic defences; (iv) metal fortifications, induced either directly or indirectly through ROS/RNS, with cell wall lignification providing a mechanical barrier against pathogens, as well as the induction of antioxidants and defence genes (Choudhury *et al.*, 2017; Terrón-Camero *et al.*, 2019), and (v) possible elemental defences, which enable metals to directly protect the plant against pathogens (Michaud and Grant, 2003; Coleman *et al.*, 2005; Matyssek *et al.*, 2005).

As explained earlier in the section “Crosstalk between plant responses to abiotic and biotic stress”, signal transduction routes in plant responses to biotic and abiotic stresses, particularly those caused by heavy metals (Romero-Puertas *et al.*, 2019), show several interaction points, mainly for short-term responses. MAPK signalling mechanisms, which are involved very early on in plant responses to various heavy metals such as Cu and Cd, differentially activate signalling routes (Suzuki *et al.*, 2001; Jonak *et al.*, 2004; Opdenakker *et al.*, 2012; Cuypers *et al.*, 2016). Extensive data are available on plant hormone responses to heavy metal stress (reviewed in Cuypers *et al.*, 2016; Anwar *et al.*, 2018; Demecsová and Tamás, 2019; Sharma *et al.*, 2020; Betti *et al.*, 2021). For example, ET signalling and biosynthesis are induced in both early and late responses to Cd in Arabidopsis (Herbette *et al.*, 2006; Weber *et al.*, 2006; Rodríguez-Serrano *et al.*, 2009).The phytohormone JA is induced by Cd and Cu stress in various plant species, such as rice, Arabidopsis, pea, and *Phaseolus coccineus* (Maksymiec *et al.*, 2005; Rodríguez-Serrano *et al.*, 2006; Ogawa *et al.*, 2009). Despite being associated with GSH and phytochelatins (Xiang and Oliver, 1998), JA is involved in the activation by metal toxicity of H_2_O_2_ production via lipoxygenase (Maksymiec *et al.*, 2005). SA, another phytohormone associated with plant responses to heavy metals, displays variable dynamics depending on the tissue and the experimental conditions (Rodríguez-Serrano *et al.*, 2009), and also affects H_2_O_2_ levels (Tao *et al.*, 2013).

Tolerance to both heavy metals and biotic stress has long been a topic of research. Several studies show that ROS metabolism and/or the induction of defence signalling pathways are involved in heavy metal protection, although the mechanisms underlying these cross-tolerance processes are sometimes unclear. Changes in the expression of cytochrome P450 genes are commonly found in the responses of Arabidopsis to Cu, as well as to *A. alternata* and *A. brassicicola*, suggesting that heavy metals induce ROS signals that serve to enhance plant resistance to fungi (Narusaka *et al.*, 2004). Pepper plants pre-treated with Cu show a phenotype that is more resistant to *Verticillium dahliae* Kleb. than plants grown under normal conditions (Chmielowska *et al.*, 2010). This resistance could be partly due to the induction of peroxidase and defence genes such as *PR1* and *β-1,3-glucanase* by treatment with Cu (Chmielowska *et al.*, 2010). Interestingly, a positive feedback loop between H_2_O_2_, Ca^2+^, and the TF WRKY41 coordinates pepper responses to *Ralstonia solanacearum* and Cd exposure (Dang *et al.*, 2019). Cu, which decreases pathogenic disease symptoms and is even used as a fungicide (Molina *et al.*, 1998), induces an increase in sensitivity in a small number of interactions (Evans *et al.*, 2007). Aluminium (Al) stress induces H_2_O_2_ accumulation and activates SA- and NO-dependent signalling pathways, which correlates with a reduction in disease symptoms in susceptible potato plants infected with *Phytophthora infestans* (Arasimowicz-Jelonek *et al.*, 2014). Interestingly, Arasimowicz-Jelonek *et al.* (2014) found that treatment with Al induces signalling mechanisms in distal tissue that are effective in combating biotic stress. Furthermore, *Vitis vinifera* pre-treated with Mn shows resistance to *Uncinula necato* due to the induction of SA, ABA, peroxidases, and defence proteins such as phenylalanine ammonia lyase, PR proteins, and an NBS-LRR analogue (Yao *et al.*, 2012).

### Metal hyperaccumulation and defence responses

Metal hyperaccumulation, defined as the capacity of some plants to accumulate abnormally high levels of a metal in the aerial parts without causing phytotoxic damage, is not very common (Poschenrieder *et al.*, 2006; Krämer, 2010; van der Ent *et al.*, 2013). Only approximately 700 taxa from distantly related families have been described as hyperaccumulators (Calabrese and Agathokleous, 2021). One hypothesis used to explain metal hyperaccumulation by plants is that metals can efficiently provide elemental defence against herbivores and pathogens (Poschenrieder *et al.*, 2006; Rascio and Navari-Izzo, 2011; Fones *et al.*, 2019). A well-documented example of this is the hyperaccumulation by *Noccaea* (formerly *Thlaspi*) *caerulescens* of zinc (Zn), whose toxicity is capable of reducing *P. syringae* pv. *maculicola* (*Psm*) growth (Fones *et al.*, 2010). In addition, while *N. caerulescens* lacks a ROS- and SA-dependent signalling capacity in response to *Psm*, Zn can induce an increase in O_2_^.−^ production in non-threatened plants (Fones *et al.*, 2013). The typical oxidative burst defence responses are shut down in *N. caerulescens* in response to *Psm*, probably due to its ability to use Zn for defensive purposes (Fones *et al.*, 2013). In fact, trade-offs between Zn tolerance and defence gene expression have also been described in relation to two *N. caerulescens* ecotypes (Plessl *et al.*, 2010). Hyperaccumulation of Zn also replaces SA- and JA-dependent defence responses in *N. caerulescens* plants threatened by *A. brassicicola* (Gallego *et al.*, 2017). *Noccaea praecox*, a Cd hyperaccumulator, is more sensitive to the powdery mildew pathogen *Erysiphe cruciferarum* at lower Cd concentrations, and low Cd supply also appears to prevent a pathogen-dependent increase in SA (Llugany *et al.*, 2013). In a similar study, the nickel (Ni) hyperaccumulator *Noccaea goesingense*, which has higher SA content than the non-accumulators Arabidopsis and *Noccaea arvense*, showed greater sensitivity to *E. cruciferarum* infection and was unable to induce SA production following infection; this sensitivity to the pathogen is reduced by Ni hyperaccumulation (Freeman *et al.*, 2005). Recent analyses of four *N. caerulescens* populations with different Zn accumulation capacities have shown that this species has different modes of action, such as metal toxicity, glucosinolate production, and cell death, in response to *Psm*, leading to trade-offs and synergistic interactions that protect the plant. Metal availability appears to be one of the factors that triggers defence responses in this case (Fones *et al.*, 2019). Trade-offs between glucosinolates and metal accumulation have also been described in relation to *Streptanthus polygaloides* and *N. caerulescens* when Ni and Cd are hyperaccumulated (Davis and Boyd, 2000; Asad *et al.*, 2013). However, the complex relationship between metal accumulation and glucosinolates may depend on the hyperaccumulator species and may even vary between specific populations (Fones *et al.*, 2019). Other factors, such as hormones and ROS, are also involved in the relationship between glucosinolates and metal accumulation, enabling hyperaccumulator plant defences to be fine-tuned, with an additional stage of regulation leading to possible joint effects that could explain hyperaccumulation (Rascio and Navari-Izzo, 2011; Kusznierewicz *et al.*, 2012; Hörger *et al.*, 2013; Gallego *et al.*, 2017). Therefore, some evidence shows that hyperaccumulated metals contribute to plant defences in the case of at least some kinds of pathogens and herbivores (Cabot *et al.*, 2019). However, the trade-offs and synergistic interactions between other signalling molecules, and how selection for resistance to disease relates to the environment during their evolution, are little understood (Hörger *et al.*, 2013).

## Cadmium and fungi: a case study

The heavy metal Cd is a non-essential element for life (Ismael *et al.*, 2019; Zhang and Reynolds, 2019) and, at even low concentrations, is toxic to living organisms (Li *et al.*, 2019; Zhang and Reynolds, 2019). Although Cd is not abundant in the earth’s crust (0.08–0.1 ppm), Cd concentrations in soils have been increasing over the past 100 years due to human activity (Rudnick and Gao, 2003; Gupta and Sandalio, 2012; Cullen and Maldonado, 2013). However, a report by the European Environment Agency (2018) shows a decrease in Cd emissions of ~64% between 1990 and 2016, mainly due to a decrease in Cd concentrations in agricultural processes and waste. Nevertheless, in 2017, the Agency for Toxic Substances and Disease Registry (http://www.atsdr.cdc.gov/) considered Cd to be the seventh most toxic heavy metal due to its toxicity and potential exposure of humans. The principal sources of Cd emissions are industrial energy consumption (29%), industrial processes and product use (28%), and the commercial, institutional and household sector (21%; European Environment Agency 2018).

Cd, which affects different ecosystems, causes atmospheric, terrestrial, and marine damage (Pinto *et al.*, 2004; Gupta and Sandalio, 2012; Li *et al.*, 2019). Following uptake by plant roots, Cd moves through the vascular bundles to other organs, including edible parts of the plant. Thus, by entering the food chain, Cd constitutes a human health hazard (Nawrot *et al.*, 2006; Liu *et al.*, 2010; Clemens *et al.*, 2013). The type II oxidation capacity and electronegativity of Cd mainly explain its toxic nature; it can form complexes with a wide variety of ligands, mainly with weak donors such as sulfide, nitrogen, and selenium (Salt and Wagner, 1993; Ismael *et al.*, 2019). One major toxic effect of Cd is redox imbalance due to disturbances of the antioxidant system, damage to the respiratory chain, and the induction of Fenton-type reactions (Cuypers *et al.*, 2016; Romero-Puertas *et al.*, 2019). Interestingly, one of the gene categories found in transcriptomic analyses of plant responses to Cd includes biotic stress responses, particularly to fungi, although little is known about crosstalk in the plant responses to Cd and fungal infections.

Pathogenic fungal microorganisms, which have been classified according to their mode of action, use a diverse range of mechanisms to infect plants. Necrotrophic pathogens use ROS/RNS, toxins, and cell-wall-degrading enzymes, among other mechanisms, to obtain nutrients from dead tissues (Wolpert *et al.*, 2002; Martínez-Medina *et al.*, 2019). Some necrotrophic pathogens even induce the overproduction of NO to accelerate infection (van Baarlen *et al.*, 2004; Sarkar *et al.*, 2014; Floryszak-Wieczorek and Arasimowicz-Jelonek, 2016), which, depending on the intensity and timing of NO production, can activate plant defences (Asai and Yoshioka, 2009). Plants also activate other signalling pathways, such as JA- and ET-dependent signalling, to activate the expression of defence-related genes (Thomma *et al.*, 2001; Kunkel and Brooks, 2002; Broekaert *et al.*, 2006). Other phytohormones, such as gibberellins, play a key role in resistance to necrotrophic pathogens due to a degraded DELLA repressor, which activates plant growth (Achard *et al.*, 2008) and interacts with a JA signalling repressor (Zhang *et al.*, 2017). Biotrophic fungal pathogens, which usually have a specific host, can induce effectors capable of suppressing plant immunity (Perfect and Green, 2001). In addition, fungi get their nutrients from living cells by maintaining host viability through specialized structural and biochemical relations (Gebrie, 2016). In some cases, fungi synthesize plant cytokinins to attract nutrients from the plant to infected tissues and to decrease the plant production of SA, thus activating plant defence biotrophic fungal genes (Choi *et al.*, 2011; Zhang *et al.*, 2017).

Conversely, plants develop mechanisms to resist biotrophic fungal infections. These include a penetration resistance mechanism, which strengthens the cell wall and membrane to halt spore germination and to prevent the formation of haustoria. Plants can also activate programmed cell death accompanied by a ROS and NO burst, leading to a hypersensitive response in penetrated epidermal cells, to shut down the supply of nutrients to the fungus (Koeck *et al.*, 2011). All of these plant defence signalling mechanisms could be points of crosstalk in plant responses to Cd and fungal pathogens; in fact, various studies have found that Cd treatments protect against fungal infections. For example, the induction of resistance to *Fusarium oxysporum* in *Triticum aestivum* by pre-treatment with Cd is related to GSH-induced glutathionylation, which protects proteins against oxidative damage (Mittra *et al.*, 2004; Mohapatra and Mittra, 2017). In addition, ROS production and cell death decrease in Cd-treated *Cajanus cajan* which was further infected with *Fusarium incarnatum*, although this was not always associated with an increase in the antioxidant system (Satapathy *et al.*, 2012). In Arabidopsis plants, increased resistance to *B. cinerea* following pre-treatment with Cd or Cu has been reported to be exclusively caused by the induction of defence genes such as *PDF1.2* (Cabot *et al.*, 2013).

### Bioinformatic analysis of the redox footprint in plant responses to Cd and fungi

The large variability in treatments, tissues analysed, culture media, plant age, and other parameters in studies conducted so far makes it difficult to reach general conclusions concerning plant responses to Cd stress. However, bioinformatic analysis provides a straightforward way to identify and analyse a common set of transcripts in plant responses to different stresses, and to identify their specificity or otherwise to different parameters, which can be very useful for future research and to better understand the mechanisms and role of these transcripts in plant responses to stress. To obtain a deeper insight into the role of ROS/RNS and redox signalling in crosstalk between plant responses to Cd and fungal pathogens, we carried out a web search of the available transcriptome analyses relating to both stresses with the aid of the PubMed (https://www.ncbi.nlm.nih.gov/pubmed/), Gene Expression Omnibus (GEO) (https://www.ncbi.nlm.nih.gov/geo/), Recursos Científicos https://www.recursoscientificos.fecyt.es/), and Scopus https://www.scopus.com/home.uri) databases. When probe information for a dataset was available, no additional filters were applied, thus ensuring that data originally filtered by the authors were used. In five studies, the differentially expressed probe lists were acquired by reanalysing the data stored in GEO. We used the GEO2R web tool (http://www.ncbi.nlm.nih.gov/geo/info/geo2r.html) with default options for differential analysis and gene list acquisition [false discovery rate (FDR) <0.05; fold change (FC) >2.0]. The search was narrowed to *A. thaliana*, which is a model plant with a larger number of available analyses, in response to Cd and a diverse range of fungi, such as *F. oxysporum*, *Fusarium graminearum*, and *B. cinerea;* these pathogens, which can infect over 150 economically important crops, are responsible for one of the highest reductions in crop productivity (Dean *et al.*, 2012). We analysed 19 microarray/RNA-seq datasets from eight different studies related to *A. thaliana* responses to Cd (Table 1), and 12 datasets from five studies of responses to fungi (Table 2).

**Table 1.** Summary of transcriptomes related to plant responses to Cd, where expression profiles of genes involved in ROS/RNS and redox-related categories were analysed using bioinformatics

Abiotic stress	Heavy metal		Plant			Expression gene analysis		Reference
ID	Concentration	Timing	Species	Tissue	Culture condition	Type	Threshold	
Cd_S_L_1 (a, b ,d, e)	5, 50 μM CdSO_4_	2, 6, 30 h	*A. thaliana*	Roots and leaves	Sand + Hydroponic, specific NS (3–4 w)	CATMA array	Bonferroni *P* value of 5%	Herbette *et al.*, 2006
Cd_L_L_1 (c, f)								
Cd_S_R_1 (g, h, j, k)								
Cd_L_R_1(i, l)								
Cd_S_R_2	50 μM Cd^2+^	2 h	*A. thaliana*	Roots	Hydroponic, Hoag. (5 w)	Affymetrix chip	*P* adj ≤0.05	Weber *et al.*, 2006
Cd_L_R_3	15 μM CdSO_4_	7 d	*A. thaliana*	Roots	Hydroponic, mod. Hoag. (3 w)	Microarray (Agilent)	FDR <0.05, FC ≥2	van de Mortel *et al*., 2008
Cd_L_R_4	15, 30 μM + 30 μM CdSO_4_	24 h	*A. thaliana*	Roots	Hydroponic, specific NS (5 w)	CATMA array	Bonferroni *P* value of 5%	Besson-Bard *et al.*, 2009
Cd_L_R_5	15 μM CdCl_2_	24 h	*A. thaliana*	Roots	MGRL medium (10 d)	Microarray (Agilent)	FC >2.5 %	Zhao *et al.*, 2009
Cd_L_C_6	10 mM CdCl_2_	12–24 h	*A. thaliana*	Cell culture	MS plates + supplements (subculture + 5 d)	CATMA array	Bonferroni *P* value <0.05	Sormani *et al.*, 2011
Cd_L_P_7	2 μM CdCl_2_	7 d	*A. thaliana*	Plant	Hydroponic, Hoag. (5 w)	Affymetrix chip	*P* adj ≤0.05	Fischer *et al.*, 2017
Cd_L_P_8	50 μM CdCl_2_	12 d	*A. thaliana*	Plant	MS plates + sucrose 1.5% (6 d)	RNA-seq	FDR <0.05	Zhou *et al.*, 2017
Cd_L_R_9	50 μM CdCl_2_	3 d	*O. sativa* cv. Huanghuazhan	Roots	Hydroponic, Kimura BNS (30 d)	RNA-seq	FDR <0.01, FC ≥2.0	Huang *et al.*, 2019
Cd_L_L_10	75 μM CdCl_2_	7 d	*O. sativa* cv. *NO. 39* Zhangzao	Leaves	Hydroponic (3 w)	RNA-seq	*P* value <0.05	Sun *et al.*, 2019
Cd_L_P_11 (a–b)	10, 100 μM CdCl_2_	24 h	*O. sativa* ssp. *japonica* cv. Nipponbare	Plant	Hydroponic, Kimura B NS (15 d)	RNA-seq	PD ≥0.2, FDR <0.05	Ye *et al.*, 2019

**Fig. 2.**
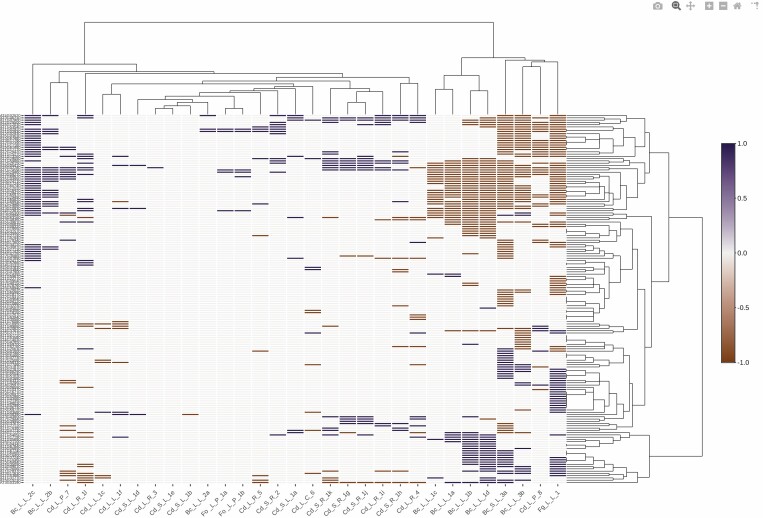
Bioinformatic analysis of the expression profile of genes involved in ROS/RNS and redox categories from Arabidopsis. Bioinformatic analysis of genes in Table S1 at Zenodo related to ROS/RNS and redox categories from Arabidopsis, which show changes in response to the different stresses. Gene up-regulation and down-regulation are indicated in blue and brown, respectively. Data were obtained from plant responses to Cd and fungal pathogen stresses described in Tables 2 and 3. Unbiased hierarchical clustering showed two clusters, I and II. Genes from groups A and B (both framed in red) were differentially regulated in clusters I and II. The code for each study (shown at the bottom) is represented by the metal or pathogen used and is described in Tables 2 and 3.

The code of each paper appears in the first column and in the abscissa axis of Figs 2, 4 and 5. The main conditions used in each paper have been summarized as metal used (Cd); time of treatment (S, short, <6 h; L, long, >6 h); tissue used (L, leaves; P, plant; R, root; S, sheath; C, cell culture); number of the paper in chronological order. For Herbette *et al.*: Cd_S_L_1a (5 μM, 2 h); Cd_S_L_1b (5 μM, 6 h); Cd_L_L_1c (5 μM, 30 h); Cd_S_L_1d (50 μM, 2 h); Cd_S_L_1e (50 μM, 6 h); Cd_L_L_1f (50 μM, 30 h); Cd_S_R_1g (5 μM, 2 h); Cd_S_R_1h (5 μM, 6 h); Cd_L_R_1i (5 μM, 30 h); Cd_S_R_1j (50 μM, 2 h); Cd_S_R_1k (50 μM, 6 h); Cd_L_R_1l (50 μM, 30 h). For Ye *et al.*: Cd_L_P_12a (10 μM), Cd_L_P_12b (100 μM). adj, adjusted; d, days; h, hours; Hoag., Hoagland solution; NS, nutrient solution; PD, percentage difference; w, weeks.

**Table 2.** Summary of transcriptomes related to plant responses to fungal pathogens where expression profile of genes involved in ROS/RNS and redox-related categories were analysed using bioinformatics

Biotic stress	Fungus		Plant			Expression gene analysis		Reference
ID	Species	Timing	Species	Tissue	Culture condition	Type	Threshold	
Fo _L_P_1 (a–b)	*F. oxysporum* (1×10^6^ spores ml^–1^)	1, 6 dpi	*A. thaliana*	Plant	MS+ sucrose 3% (2 w)	RNA-seq	RPKM >1	Zhu *et al.*, 2013
Fg_L_L_1	*F. graminearum* (1×10^5^ spores ml^–1^)	3 dpi	*A. thaliana*	Leaves	Soil (flowering plants)	Microarray (Agilent)	*P* adj <0.05, –1>log_2_FC >1 *	Miwa *et al.*, 2017
Bc_L_L_1 (a–d)	*B. cinerea* (5×10^4^ spores ml^–1^)	18, 22 hpi	*A. thaliana*	Leaves	Soil (4 w)	Microarray (NimbleGen)	*P* adj <0.05, –1>log_2_FC>1 *	Ingle *et al.*, 2015
Bc_L_L_2 (a–c)	*B. cinerea* (1×10^5^ spores ml^–1^)	12, 18, 24 hpi	*A. thaliana*	Leaves	River sand+ Hoag. (4–5 w)	RNA-seq	FDR <0.05, –1>log_2_FC>1	Coolen *et al.*, 2016
Bc_S_L_3 (a–b)	*B. cinerea* (1–5×10^5^ spores ml^–1^)	6, 48 hpi	*A. thaliana*	Leaves	Soil (4 w)	Microarray (Agilent)	*P* adj <0.05, –1>log_2_FC>1 *	Wang *et al.*, 2018
Mo_L_S_1	*M. oryzae* (1×10^5^ spores ml^–1^)	36 hpi	*O. sativa*	Sheath	Soil (3 w)	Microarray (Agilent)	FC >50, *P*<2.2× 10^6^	Mosquera *et al.*, 2009
Mo_L_L_2 (a–d)	*M. oryzae* (1×10^5^ spores ml^–1^)	1, 2 dpi	*O. sativa* L. cv. LTH (compatible), IRBL1 (incompatible)	Leaves	Soil (2 w)	Microarray (Agilent)	*P* logratio >0.05, 0.9<FC<1.2	Kato *et al.*, 2009
Mo_L_L_3	*M. oryzae* (1×10^5^ spores ml^–1^)	2 dpi	*O. sativa* L. cv. Nipponbare	Leaves	Soil (2 w)	Microarray (Agilent)	*P* adj <0.05, –1>log_2_FC>1 *	Chujo *et al.*, 2013
Mo_L_L_4 (a-h)	*M. oryzae* (1×10^5^ spores ml^–1^)	1, 2, 3, 5 dpi	*O. sativa* cv. Nipponbare NP/++ (compatible), NP/Pia (incompatible)	Leaves	Hydroponic, specific NS (2 w)	Microarray (Agilent)	*P* adj <0.05, –1>log_2_FC>1 *	Tanabe *et al.*, 2014
Mo_L_L_5	*M. oryzae* (1×10^5^ spores ml^–1^)	2 dpi	*O. sativa* cv. Tainung67, *japonica*	Leaves	Soil (3–4 leaves stage)	RNA-seq	FDR <0.05, –1<log_2_FC<1	Sánchez-Sanuy *et al.*, 2019

The code of each paper appears in the first column and in the abscissa axis of Figs 2, 4 and 5. The main conditions used in each paper have been summarized as fungi (Fo: *Fusarium oxysporum*, Fg: *Fusarium graminearum*, Bc: *Botrytis cinerea*; Mo: *Magnaporthe oryzae*); time of the treatment (S, short, <6 h; L, long, >6 h); tissue used (L, leaves; P, plant; R, root; S, sheath; C, cell culture); number of the paper by chronological order. For Zhu *et al.*: Fo _L_P_1a (1 dpi); Fo_L_P_1b (6 dpi). For Ingle *et al.*: Bc_L_L_1a (D 18 dpi); Bc_L_L_1b (D 22 dpi); Bc_L_L_1c (N 18 dpi); Bc_L_L_1d (N 22 dpi). For Coolen *et al.*: Bc_L_L_2a (12 hpi); Bc_L_L_2b (18 hpi); Bc_L_L_2c (24 hpi). For Wang *et al.*: Bc_S_L_3a (6 h); Bc_L_L_3b (48 h). For Kato *et al.*: Mo_L_L_2a (comp, LTH-24 h), Mo_L_L_2b (comp LTH-48 h), Mo_L_L_2c (incomp IRBL-24 h), Mo_L_L_2d (incomp IRBL-48 h). For Tanabe *et al.*: Mo_L_L_4a (1 d incomp), Mo_L_L_4b (2 d incomp), Mo_L_L_4c (3 d incomp), Mo_L_L_4d (5 d incomp), Mo_L_L_4e (1 d comp), Mo_L_L_4f (2 d comp), Mo_L_L_4g (3 d comp), Mo_L_L_4h (5 d comp). dpi, days post infection; hpi, hours post infection; w, weeks. Asterisks indicate data analysed for this review by using the GEO2R web tool (http://www.ncbi.nlm.nih.gov/geo/info/geo2r.html).

The shortage of crop species data in some cases and barely identified transcripts in others, as well as the variability in the nomenclature used to define genes, are major barriers to carrying out bioinformatic meta-analysis. We used rice (*Oryza sativa* L.), one of the most important cereal crops, as a model monocotyledonous plant, although only 25% of the data published could be analysed in our meta-analysis. Rice, which is the principal food for almost half of the world’s population, is usually grown in paddy fields under flood conditions, and is therefore more susceptible to heavy metals contamination (Sun *et al.*, 2019). We identified four different profile analyses in three studies of rice responses to Cd and 15 profile analyses in five studies of rice responses to *Magnaporthe oryzae*, which causes blast disease and seriously affects rice yields (Sánchez-Sanuy *et al.*, 2019) (Table 1 and Table 2).

Expression profiles of genes involved in ROS/RNS and redox-related categories according to the Gene Ontology (GO) resource (http://geneontology.org/) (Table 3) were analysed in the transcriptomes described in Tables 1 and 2. These categories include 210 genes in *A. thaliana* and 218 genes in *O. sativa* (see Table S1 at Zenodo Repository, https://zenodo.org/record/5040382#.YNrth5j7S71). A total of 82 RBOHD- and H_2_O_2_-dependent genes in systemic responses to different stress conditions have also been analysed (Zandalinas *et al.*, 2019). Probes were annotated with locus identifiers using the TAIR Microarray Elements Search and Download tool for *A. thaliana* or were converted to ORF IDs using the UniProt (https://www.uniprot.org/) and NCBI GPL19274 (https://www.ncbi.nlm.nih.gov/geo) databases for *O. sativa*. All probes were then categorized under the following headings: no data/no change, increase, and decrease. After the first analysis, genes not expressed in any treatment were removed and the selected data were reanalysed. We then performed a hierarchical clustering analysis to objectively search for groups of probes in an unsupervised manner without specifying the number of clusters to be created. We used H-clustering, heatmaply, and htmlwidgets in the R software package to do this.

**Table 3.** Summary of ROS/RNS and redox-related categories analysed using bioinformatics in Figs 2, 4, and 5

Category	GO code
*S*-nitrosoglutathione reductase activity	GO:0080007
Response to redox state	GO:0051775
l-methionine:thioredoxin-disulfide *S*-oxidoreductase activity	GO:0033744
Peroxiredoxin activity	GO:0051920
Thioredoxin-disulfide reductase activity	GO:0004791
Thioredoxin peroxidase activity	GO:0008379
Cell redox homeostasis	GO:0045454
Cellular response to redox state	GO:0071461
Detection of redox state	GO:0051776
Antioxidant activity	GO:0016209
Glutathione peroxidase activity	GO:0004602
Glutathione transferase activity	GO:0004364
Glutathione metabolic process	GO:0006749
l-ascorbate peroxidase activity	GO:0016688
Monodehydroascorbate reductase (NADH) activity	GO:0016656
Hydrogen peroxide mediated signalling pathway	GO:0071588
Response to hydrogen peroxide	GO:0042542
Response to superoxide	GO:0000303

#### Arabidopsis thaliana

**Fig. 3.**
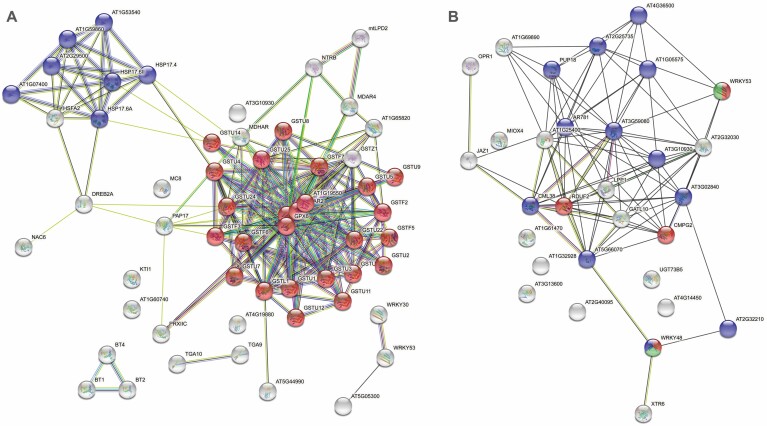
Enrichment analysis of genes from groups A and C. (A) String analysis (https://string-db.org/) of genes from group A (see Fig. 2) related to ROS/RNS and redox metabolism and differentially regulated in clusters I and II. These genes showed one main group related to glutathione metabolism (in red), the strongest KEGG pathway, and a smaller group related to protein processing in the endoplasmic reticulum (in blue), as described in Table S2 at Zenodo. (B) String analysis of genes from group C (see Fig. 4) related to systemic RBOHD- and H_2_O_2_-dependent transcripts from Arabidopsis and differentially regulated in clusters I and II. These genes showed one main group related to responses to chitin (in red) and responses to chitin, as well as the cysteine-rich transmembrane (CYSTM) domain (in blue), the strongest KEGG pathway, as described in Table S2 at Zenodo.

When analysing genes involved in ROS/RNS and the redox category (Table 3; Fig. S1 at Zenodo Repository, https://zenodo.org/record/5040382#.YNrth5j7S71), a group of *A. thaliana* genes that showed no changes in response to any of the stresses examined was removed. Further clustering analysis enabled us to find two clusters (I and II) for the stresses applied based on the induction or repression, respectively, of a group of 57 genes (group A; Fig. 2; Fig. S2, Table S2 at Zenodo Repository, https://zenodo.org/record/5040382#.YNrth5j7S71). Cluster I mainly involves the fungal pathogens *B. cinerea* and *F. graminearum* in plants growing in soil and the Cd treatment Cd_L_P_8, the longest treatment analysed (12 days) (Fig. 2). Cluster II involves most of the Cd treatments, *F. oxysporum*, and one study of *B. cinerea* with plants growing in sand supplemented with Hoagland solution. String analysis of these group A genes showed one main group, related to glutathione metabolism, to be the strongest KEGG pathway (Fig. 3A; Table S2 at Zenodo Repository, https://zenodo.org/record/5040382#.YNrth5j7S71), as well as genes associated with ASC metabolism, particularly those encoding dehydro- and monodehydro-ascorbate reductases. As H_2_O_2_ has been shown to be directly related to glutathione status, different H_2_O_2_-dependent signalling pathways may be regulated by GSH (Noctor *et al.*, 2012). Given its chemical properties, glutathione, which can undergo different redox reactions, is a key molecule involved in the regulation of the cellular redox network (Noctor *et al.*, 2012).

Genes related to glutathione metabolism from group A mainly include glutathione *S*-transferases (GSTs) and two glutathione peroxidases. GSTs are a diverse group of multi-functional proteins essential for protecting plants against oxidative damage, in what has been classified as a phase II detoxification system (reviewed in Gullner *et al.*, 2018). GSTs catalyse the conjugation of GSH to a variety of electrophilic and hydrophobic substrates, including xenobiotic compounds, which are then sequestered in vacuoles to prevent substrate toxicity. GSTs are also involved in removing excess lipid hydroperoxides produced in response to stress (Gullner *et al.*, 2018). Plant GSTs have been categorized into four classes: phi, tau, lambda, and dehydroascorbate reductase GSTs (Edwards and Dixon, 2005). Although the precise metabolic functions of GST isoenzymes in plant infection and abiotic stress have not been determined, their most important role, acting as glutathione peroxidases, could be to affect lipid hydroperoxides. GST transcripts have been reported to be up-regulated in response to stress conditions, such as fungal or bacterial infection (reviewed in Gullner *et al.*, 2018), heavy metals, cold, salt, H_2_O_2_, UV, and light (reviewed in Kumar and Trivedi, 2018). However, their single-/multiple-stress responsiveness or possible redundant functions depend on the class of GSTs to which they belong (Sappl *et al.*, 2009). We have identified a group of genes that are regulated under Cd treatment and fungal infection regardless of a wide range of experimental conditions. The induction of a group of GST-encoding genes suggests that the induction of Cd-stress-related genes could provide protection against fungal infection.

Following string analysis, a smaller number of genes from group A were also grouped together on the basis of protein processing in the endoplasmic reticulum (ER) (Fig. 3A; Table S2 at Zenodo Repository, https://zenodo.org/record/5040382#.YNrth5j7S71) and, in particular, of ER-associated degradation (ERAD); this subgroup of genes encoded heat shock proteins. ERAD is involved in the degradation of terminally misfolded proteins. In fact, in Arabidopsis plants, low concentrations of ROS, acting as signalling molecules, have been shown to induce ER stress-related genes, whose regulation is dependent on the compartment from which the ROS originated, such as the chloroplasts, mitochondria, and peroxisomes (Ozgur *et al.*, 2015). In our study, ERAD cluster I genes were repressed mainly by *B. cinerea* and long-term Cd treatment, while cluster II genes were induced. Repression of ERAD may induce ER stress, which activates signalling pathways or unfolded protein responses involved in ER protection, which, when insufficient to restore ER function, can lead to cell death by apoptosis.

Group B, containing 23 probes (Table S2 at Zenodo Repository, https://zenodo.org/record/5040382#.YNrth5j7S71), was induced in cluster I, but, unlike group A, no changes or distinct types of induction were observed in cluster II (Fig. 2). String analysis of group B did not show any clear interacting groups, although the genes involved appear to be mainly related to the glutathione metabolism by GSTs and to antioxidant-detoxification processes (Table S2 at Zenodo Repository, https://zenodo.org/record/5040382#.YNrth5j7S71). Our results show that both groups A and B were mainly related to genes encoding GSTs, with specific footprints being observed in both clusters. As described above, our experimental results indicate the important role played by these genes in plant protection against Cd and fungal stresses, as has previously been described with respect to wheat and *F. oxysporum* (Mittra *et al.*, 2004; Mohapatra and Mittra, 2017). Therefore, glutathione metabolism, and particularly the GST-related metabolism, may be key players in the crosstalk between heavy metal and fungal pathogen stress responses. In fact, Arabidopsis mutants overexpressing GSTs show higher tolerance to fungal infection (Gullner *et al.*, 2018) and to various abiotic stresses such as heavy metals, cold, and salt (Kumar and Trivedi, 2018).

**Fig. 4.**
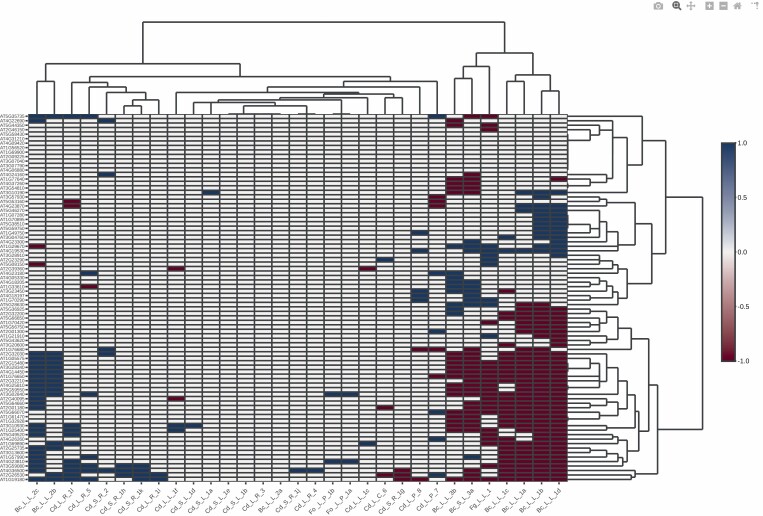
Bioinformatic analysis of the expression profile of systemic RBOHD- and H_2_O_2_-dependent transcripts from Arabidopsis. Bioinformatic analysis of genes from Zandalinas *et al.* (2020) related to systemic RBOHD- and H_2_O_2_-dependent transcripts. Gene up-regulation and down-regulation are indicated in blue and brown, respectively. Data were obtained from plant responses to Cd and fungal pathogen stresses described in Tables 2 and 3. Unbiased hierarchical clustering showed two clusters, I and II. Genes from group C (framed in red) were differentially regulated in clusters I and II. The code for each study (shown at the bottom) is represented by the metal or pathogen used and is described in Tables 2 and 3.

When analysing systemic RBOHD- and H_2_O_2_-dependent transcripts, we also found two clusters (I and II) corresponding to a group of 30 genes (group C) that were induced or repressed, respectively, under the stresses applied (Fig. 4; Fig. S3, Table S2 at Zenodo Repository, https://zenodo.org/record/5040382#.YNrth5j7S71). Clusters in this analysis were similar to those previously analysed except for the Cd_L_P_8 treatment, which is now included in cluster II with all the other Cd treatments. String analysis of the 30 group C genes found a main group based on the biological process: response to chitin (Fig. 3B, Table S2 at Zenodo Repository, https://zenodo.org/record/5040382#.YNrth5j7S71). Perception of fungal pathogens by the plant occurs through the recognition of chitin, a polymer component of the fungal cell wall, followed by the activation of the plant immune response (Squeglia *et al.*, 2017). Our bioinformatic analysis showed that gene group C is down-regulated in cluster II, which is mostly composed of *B. cinerea* treatments. The process of infection by *B. cinerea* includes an initial production of local necrotic lesions followed by lesion spreading at a later stage (Bi *et al.*, 2021), suggesting that the plant response to the pathogen is repressed. Cd-induced genes related to responses to chitin may help to protect plants against fungal infection following Cd treatment, a process that requires further exploration. Interestingly, different plant culture conditions may affect the expression of the group C genes, as *B. cinerea* with plants cultured in river sand supplemented with Hoagland solution, as well as *F. oxysporum* with plants cultured in Murashige and Skoog medium supplemented with sucrose, showed an opposite trend in gene expression to that for fungi such as *B. cinerea* and *F. graminearum* with plants cultured in soil.

#### Oryza sativa

**Fig. 5.**
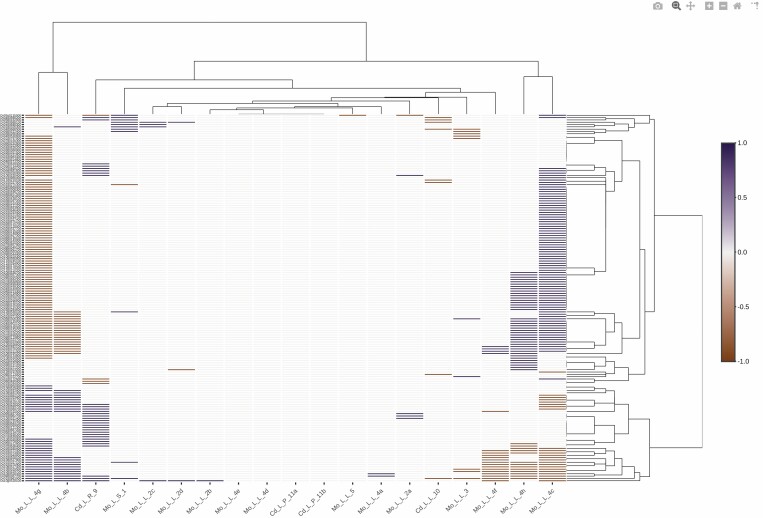
Bioinformatic analysis of the expression profile of genes involved in ROS/RNS and redox categories from rice. Genes analysed are summarized in Table S1 at Zenodo. Gene up-regulation and down-regulation are indicated in blue and brown, respectively. Data were obtained from plant responses to Cd and fungal pathogen stresses described in Tables 2 and 3. Unbiased hierarchical clustering showed two, clusters I and II. Genes from group D (framed in red) were differentially regulated in response to abiotic and biotic stresses. The code for each study (shown at the bottom) is represented by the metal or pathogen used and is described in Tables 2 and 3.

The clustering of data from *O. sativa* has been complicated, probably due to lower availability of data and the diversity of cultivars used; each transcriptomic analysis of Cd treatment was carried out with a different cultivar, and the behaviour of these different cultivars may differ under similar environmental conditions. In addition, different lines, which were either compatible or incompatible with the fungal pathogen *M. oryzae,* were analysed in the same cultivar. Despite these problems, clustering analysis of transcriptome changes in genes involved in ROS/RNS and redox categories (Table 3) in rice responses to Cd and *M. oryzae* enabled us to find two clusters (I and II) for the stresses applied, based on the induction or repression, respectively, of a number of genes (group D; Fig. 5; Fig. S4, Table S2 at Zenodo Repository, https://zenodo.org/record/5040382#.YNrth5j7S71). Cluster I involves both compatible and incompatible rice interactions *M. oryzae*, with different timings; this suggests that different induction/repression waves of redox-related genes take place during the treatment, which are associated with a type of interaction. Cluster II involves all the other treatments analysed, in most of which only a few genes underwent changes (Fig. 5). Cluster I and Cd_L_R_9 behaved similarly to a group of 32 induced genes, which were repressed in cluster II. String analysis of these genes showed no gene pooling; most of the genes were related to glutathione metabolism, the strongest KEGG pathway, mainly encoding GSTs (Table S2, Fig. S5 at Zenodo Repository, https://zenodo.org/record/5040382#.YNrth5j7S71). These results suggest that rice plants growing in Cd for short to medium periods of time may also show induction of GST activity and therefore be more resistant to fungal pathogens, similar to the findings with Arabidopsis plants and in previous studies of wheat (Mittra *et al.*, 2004; Mohapatra and Mittra, 2017).

## Conclusions and perspectives

Plant responses to certain stresses have been well characterized when applied individually, which has provided the basis for establishing models with key components involved in plant responses to stress. However, as plants are usually confronted with more than one stress in the field, we need to build similar models for serial and combined stresses, which would be unique for each combination. Combinations of abiotic and biotic stresses are of particular importance given the singular nature of each interaction between two or more organisms. Recent advances in the study of plant responses to combinations of stresses point to a role for key signalling molecules, including hormones, TFs, and, in particular, to ROS/RNS and redox homeostasis, for selecting different pathways to achieve a trade-off between acclimation/survival and yield. Bioinformatic analyses of transcriptome changes in plant responses to Cd and fungal pathogens point to redox signalling at the crossroads of both these stresses, which is mainly related to the glutathione metabolism, particularly with respect to GST genes. We identified different groups of GST genes that are up- or down-regulated depending on the treatment (Cd/fungi). The results obtained indicate that genes encoding GSTs are a key gene family in relation to a broad range of species at the crossroads of plant responses to biotic and abiotic stresses. We identified other groups of genes, such as ERAD genes associated with heat shock proteins, as well as those involved in responses to chitin, which may also be involved in crosstalk between abiotic and biotic stresses, particularly Cd and fungal infections. Our bioinformatic findings should pave the way for more comprehensive future research into crosstalk between different stresses. The characterization of the key molecules identified in different stress combinations could lead to the development of new strategies to alleviate the effects of multifactorial stress conditions, especially in the current context of global climate change.

## Data Availability

The following data are available at Zenodo Repository, https://zenodo.org/record/5040382#.YNrth5j7S71; Romero-Puertas *et al.* (2021). Complete expression profile of genes involved in ROS/RNS and redox categories from Arabidopsis; bioinformatic analysis of the expression profile of genes involved in ROS/RNS and redox categories from Arabidopsis; bioinformatic analysis of the expression profile of RBOHD- and H_2_O_2_-dependent systemic transcripts from Arabidopsis; bioinformatic analysis of the expression profile of genes involved in ROS/RNS and redox categories from rice; enrichment analysis of genes in group D; genes and GO categories used for analysis; genes from groups A to D and KEGG pathways obtained after enrichment analysis.
